# Relationship between socioeconomic status and weight gain during infancy: The BeeBOFT study

**DOI:** 10.1371/journal.pone.0205734

**Published:** 2018-11-02

**Authors:** Lu Wang, Amy van Grieken, Junwen Yang-Huang, Eline Vlasblom, Monique P. L'Hoir, Magda M. Boere-Boonekamp, Hein Raat

**Affiliations:** 1 Department of Public Health, Erasmus University Medical Center, Rotterdam, the Netherlands; 2 TNO Child Health, Leiden, the Netherlands; 3 Department of Agrotechnology and Food Sciences, Subdivision Human Nutrition, Wageningen University & Research, Wageningen, the Netherlands; 4 Department Health Technology and Services Research, Technical Medical Centre, University of Twente, Enschede, the Netherlands; Vanderbilt University, UNITED STATES

## Abstract

**Background:**

Increased weight gain during infancy is a risk factor for obesity and related diseases in later life. The aim of the present study was to investigate the association between socioeconomic status (SES) and weight gain during infancy, and to identify the factors mediating the association between SES and infant weight gain.

**Methods:**

Subjects were 2513 parent-child dyads participating in a cluster randomized controlled intervention study. Family SES was indexed by maternal education level. Weight gain in different time windows (infant age 0–3, 0–6, and 6–12 months) was calculated by subtracting the weight for age *z*-score (WAZ) between the two time-points. Path analysis was performed to examine the mediating pathways linking SES and infant weight gain.

**Results:**

On average, infants of low-educated mothers had a lower birth weight and caught-up at approximately 6 months. In the period of 0–6 months, infants with low-educated mothers had an 0.42 (95% CI 0.27–0.57) higher gain in weight for age z-score compared to children with high-educated mothers. The association between maternal education level and increased infant weight gain in the period of 0–6 months can be explained by infant birth weight, gestational age at child birth, duration of breastfeeding, and age at introduction of complementary foods. After adjusting all the mediating factors, there was no association between maternal education level and infant weight gain.

**Conclusion:**

Infants with lower SES had an increased weight gain during the first 6 months of infancy, and the effect can be explained by infant birth weight, gestational age at child birth, and infant feeding practices.

## Introduction

The high prevalence of child obesity and concomitant effects on morbidity and mortality constitute a major public health concern. [1] In developed countries, low socioeconomic status (SES) is consistently associated with a higher prevalence of childhood obesity. [2–5] Understanding the origins of socioeconomic inequalities in childhood obesity may contribute to the development of intervention programs.

Increased weight gain during infancy -in varying age windows from the first week of life to the first two years of life—has been consistently associated with childhood obesity. [6–8] In addition, increased weight gain during infancy has been associated with increased risk of cardiovascular risk factors in later life, such as high blood pressure, insulin resistance, and endothelial dysfunction. [9–14] Only two previous studies have focused on examining the association between SES and weight gain during infancy. [15, 16] These two studies suggested that lower SES is associated with increased weight gain during the first three months [15] and the first year of life. [16] In both studies, only two time points were used to assess weight gain during infancy, which may not be sufficient to capture the differences in the weight gain trajectories during infancy. [15, 16] It is not known whether the SES differences in infant weight gain is consistent during infancy. In addition, some important potential confounding factors/mediating factors were missing in these studies, such as parental height, and maternal pregnancy complications.

Previous studies have indicated that the determinants of infant weight gain include prenatal factors such as parental body mass index (BMI), parental height, gestational age, infant birth weight, and infant feeding practices such as breastfeeding duration and age at introduction of complementary feeding. [17–22] Yet, it is not clear to what extent the association between SES and infant weight gain can be explained by these determinants. To develop interventions to reduce the SES-related inequality in childhood obesity prevalence, insight in the factors that are most important for explaining the association between SES and infant weight gain is needed.

Therefore, the aim of the present study was to investigate the association between SES and weight gain during infancy, and to identify the mediating factors explaining the association between SES and infant weight gain.

## Methods

### Study design and study population

This study used data from the ‘BeeBOFT Study’, a population-based 3-armed cluster randomized trial for the primary prevention of overweight among younger children (Netherlands Trial Register: NTR1831).[23] The ‘BeeBOFT study’ has been conducted within 51 regional Youth health care (YHC) teams. Parents who were allocated to the first arm of the trial, the ‘BBOFT+’ intervention, received intervention on healthy behavioral life-style habits of the children from birth onward at each YHC routine visit (at child age 0, 1, 2, 3, 4, 6, 9, 11, 14, 24, 36, and 48 months). The ‘E-health4Uth Healthy toddler’ intervention, the second arm of the trial, provided the parents tailored health education regarding healthy child nutrition and activity behaviors at the child age of circa 18 and 24 months old. Parents in the control group received care as usual. The research proposal was reviewed by the Medical Ethics Committee of the Erasmus Medical Center. Based on their review, the Committee concluded that the Dutch Medical Research Involving Human Subjects Act (in Dutch: Wet medisch-wetenschappelijk onderzoek met mensen) did not apply to this research proposal. The Medical Ethics Committee therefore had no objection to the execution of this study (proposal number MEC-2008-250). Further details about the study design and the interventions are described in the design paper published by Raat et al.[23]

Parents were invited to participate in the ‘BeeBOFT’ study when the Youth Health Center (YHC) nurses visited them at home in the second week after child birth, between 2009 and 2010. Written informed consent to participation was gained from the parents of 3003 infants. For the present study, we only included infants with weight measurements available at birth, and at least 1 measurement at 3, 6 or 12 months’ age (n = 2552). We excluded participants with no information on maternal education level (n = 39). Eventually 2513 infants were included in the present study.

### Measurement

#### Socioeconomic status

Maternal education level was used as the main indicator of family social economic status. Other indicators of family socioeconomic status included paternal education level, and both maternal and paternal employment status. Data on maternal and paternal education level, maternal and paternal employment status were obtained from baseline parental questionnaires at the child’s age of 2–4 weeks. Following the standard definition of Statistics Netherlands,[24] the maternal highest attained education level was categorized as high (higher vocational training, university degree), middle (>3 years general secondary school or intermediate vocational training), or low (no education, primary school, or three years or less general secondary school).

#### Child growth assessments

Data on weight (and height) of the child were acquired from the YHC registration files. At each YHC routing visit (at child age of circa 0, 1, 2, 3, 4, 6, 9, 12 month), child weight and height was measured using standardized methods by YHC nurses [25]. We used child weight measurement at child age of circa 0, 3, 6, and 9 months. To adjust weight for physiological growth and gender differences, the weight-for-age *z*-scores (WAZ) were calculated using the Dutch 1997 age- and gender-specific reference values. [26] Infant weight gain in different time windows (0–3 months, 0–6 months, and 6–12 months) was assessed by changes in WAZ between the two time-points. An increase in WAZ of greater than 0.67 in each time window was defined as rapid weight gain.[7, 27]

#### Potential mediators

Potential mediators for the association between SES and infant weight gain were selected based on previous researches linking them with infant weight gain [17–22] and the rational plausibility that SES may influence these factors.

#### Infant characteristics

Infant characteristics at birth including gestational age at birth (weeks) and birth weight are highly related to the velocity of infant weight gain. Gestational age at child birth was obtained by baseline questionnaire. We created gestational age- and gender-adjusted infant birth weight (weight for gestational age z- score) within our study population based on North European growth charts.[28]

#### Prenatal factors

Prenatal factors included maternal age at child birth, maternal pre-pregnancy BMI, paternal BMI, maternal and paternal height (meters), maternal gestational weight gain (kilogram), maternal diabetes (Yes/No), maternal hypertension (Yes/No), and parity. [17, 19] Maternal pre-pregnancy BMI and paternal BMI was calculated by weight(kilogram)/ height^2^(meter). These variables were self-reported by parents in baseline questionnaire at child age 2–4 weeks.

#### Infant feeding practices

Infant feeding factors that may influence infant growth included duration of breastfeeding, and timing of introduction of complementary feeding. In the questionnaire at child age 6 months, parents were asked to report whether they have initiated breastfeeding, and how old the child was when they stopped giving breastfeeding. Parents were also asked to report how old the child was when they started to give the child foods or liquids besides breast milk or formula. The coding of breastfeeding duration and age at introduction of complementary feeding are shown in S1 Table.

#### Potential confounders

Child gender and exact age at measurement, child ethnic background (non-native VS native) were considered as potential confounders, since they can bias the association, but are not on the causal pathway between SES and infant weight gain. The child’s ethnic background was defined as native only if both parents had been born in the Netherlands.[29]

### Statistical analysis

Descriptive statistics were calculated to present sample characteristics. We plotted the average weight for age z-score trajectories between child birth and 12 months of age according to maternal education level (by R package ‘ggplot’), using all the available weight measurements in the period of 0–12 months.

Linear regression models were used to examine the association between maternal education level and infant weight gain (represented by changes in WAZ) at different times windows of infant growth (0 to 3, 0 to 6, and 6 to 12 months). Logistic regression models were used to examine the association between maternal education level and rapid weight gain (Yes vs No). The models were adjusted for potential confounders including child gender, ethnic background, and age at weight measurement. To handle the multiple testing problem, Bonferroni correction was adopted, which sets the significance level at α/n = 0.05/3 = 0.017.

We examined the independent association of the potential mediators with infant weight gain using multivariate linear regression models, adjusting for maternal educational level and potential confounders. Factors associated with both infant weight gain and maternal education level were considered as potential mediators in the following path analysis (p<0.10), [30] including: gestational age at child birth, weight for gestational age z-score, duration of breastfeeding, age at introduction of complementary feeding, parental heights, maternal BMI. A path analysis mediation model was used to examine the mediating pathways between maternal education level and infant weight gain (Proc Calis procedure in SAS). The path analysis model consisted of regressions models which a) regressed the infant weight gain on maternal education level and the potential mediators, and b) regressed the potential mediators on maternal education level. The goodness of fit index (GFI) for the path analysis model was 0.94, and the Root Mean Square Error of Approximation (RMSEA) was 0.07, suggesting a favorable model fit.[31] The indirect effect of maternal education level for each of the pathways was calculated as the product of regression coefficients on that pathway.[30, 32] The proportion of the effect of SES on infant weight gain mediated by each mediator was determined by dividing the corresponding absolute indirect effect by the total effect.[33]

Some of the potential mediators had missing values, ranging from 0.03% missing (maternal height) to 23% missing (age at introduction of complementary feeding). To reduce potential bias associated with missing data, a multiple imputation procedure was performed. As the variables with missing values included both continuous (e.g. gestational age) and categorical variables (e.g. maternal hypertension), a multiple imputation procedure by fully conditional specification (FCS) was used.[34, 35] Twenty imputed datasets were generated to represent a plausible range of values that approximate the missing values. All the potential mediators and confounders, maternal education level, and infant WAZ were used as predictors for the missing data imputation. Pooled regression coefficients are shown in the results for the multiple linear regression analyses and path analyses. To check the quality of the imputation, we inspected the distribution of the imputed variables in the completed dataset and in the original dataset (S2 Table). There were no apparent differences between the completed data and original data.

We repeated the analysis by using other SES indicators including paternal education level, paternal and maternal employment status to assess the SES gradient in infant weight gain (S3 Table). Also, we repeated the analysis using gain in weight for height z-scores, and BMI z-scores in different time windows as outcomes, the results are shown in S4 Table. We performed a sensitivity analysis using data from subjects who did not receive any intervention (the parent-child dyads who were allocated to the control group and ‘E-Health’ group), the results were comparable (S5 and S6 Tables). In addition, we performed sensitivity analysis using the complete cases with no missing values on the potential mediators, the results were comparable (S7 Table).

## Results

### Sample characteristics according to maternal education level

Table 1 shows the general characteristics of the study participants in the total study population, and by maternal education. The percentage of low-, middle-, and high-educated mothers was 13.5%, 35.4%, and 51.1% respectively. Infants with low-educated mothers had shorter gestational age, and a smaller birth weight (*p*<0.05). Numbers of male and female infants were equal. A percentage of 16.3 of the infants have a non-Dutch ethnic background. Low-educated mothers were younger at infant birth, had a shorter height, and higher pre-pregnancy BMI. With regard to infant feeding practices, lower educated mothers gave breastfeeding for a shorter duration and introduced complementary food earlier.

**Table 1:** Characteristics of the infants and parents according to maternal education level.

			Maternal education level			
	Missing	Total	Low	Middle	High	*p*-value
		2513	339	890	1284	
**Infant characteristics**						
Gender, male (%)	0	50.80	55.12	48.65	51.18	0.12
Ethnic background, non-Dutch (%)	0	16.27	19.17	14.94	16.42	0.01
Gestational age (weeks)	31	39.65(1.31)	39.51 (1.42)	39.65(1.30)	39.69(1.28)	0.01
Weight for gestational age z-score at birth	31	0.10 (0.99)	-0.12 (1.08)	0.06 (0.98)	0.18 (0.96)	<0.001
**Prenatal factors**						
Maternal age at child birth (years)	20	30.93(4.3)	29(5.3)	30.21(4.31)	31.94(3.68)	<0.001
Maternal pre-pregnancy BMI (kg/m^2^)	102	24.21(4.49)	24.79(5.25)	24.77(4.7)	23.69(4.04)	<0.001
Paternal BMI (kg/m^2^)	254	25.25 (3.3)	25.44 (4.32)	25.52 (3.49)	25.02(2.85)	0.002
Maternal height (meters)	9	1.7(0.07)	1.68(0.06)	1.69(0.07)	1.71(0.07)	<0.001
Paternal height (meters)	48	1.83(0.07)	1.81(0.08)	1.82(0.07)	1.84(0.07)	<0.001
Gestational weight gain mother (kg)	139	14.16(5.26)	13.72(5.83)	14.22(5.71)	14.23(4.75)	0.21
Maternal hypertension ^1^(%)	32	9.53	8.54	11.34	8.53	0.08
Maternal diabetes (%)	32	1.59	2.13	1.72	1.36	0.56
Parity, primipara (%)	0	46.66	50.45	44.63	47.07	0.17
**Infant feeding practices**						
Started breastfeeding after birth (%)	172	81.16	72.63	80.68	89.09	<0.001
Breastfeeding duration (months)	223	2.83(2.77)	1.42 (2.30)	2.36(2.69)	3.51(2.74)	<0.001
Age at introduction of complementary foods (months)	578	4.62(0.95)	4.07(1.1)	4.51(0.94)	4.8(0.88)	<0.001

Note: Continuous variables are presented as means (SD), and categorical variables are presented as percentage. Differences were tested with One way ANOVA analysis and Chi-square tests.

^1^: maternal hypertension disorders included maternal hypertension and eclampsia

### Maternal education level and weight gain/rapid weight gain

**Fig 1:**
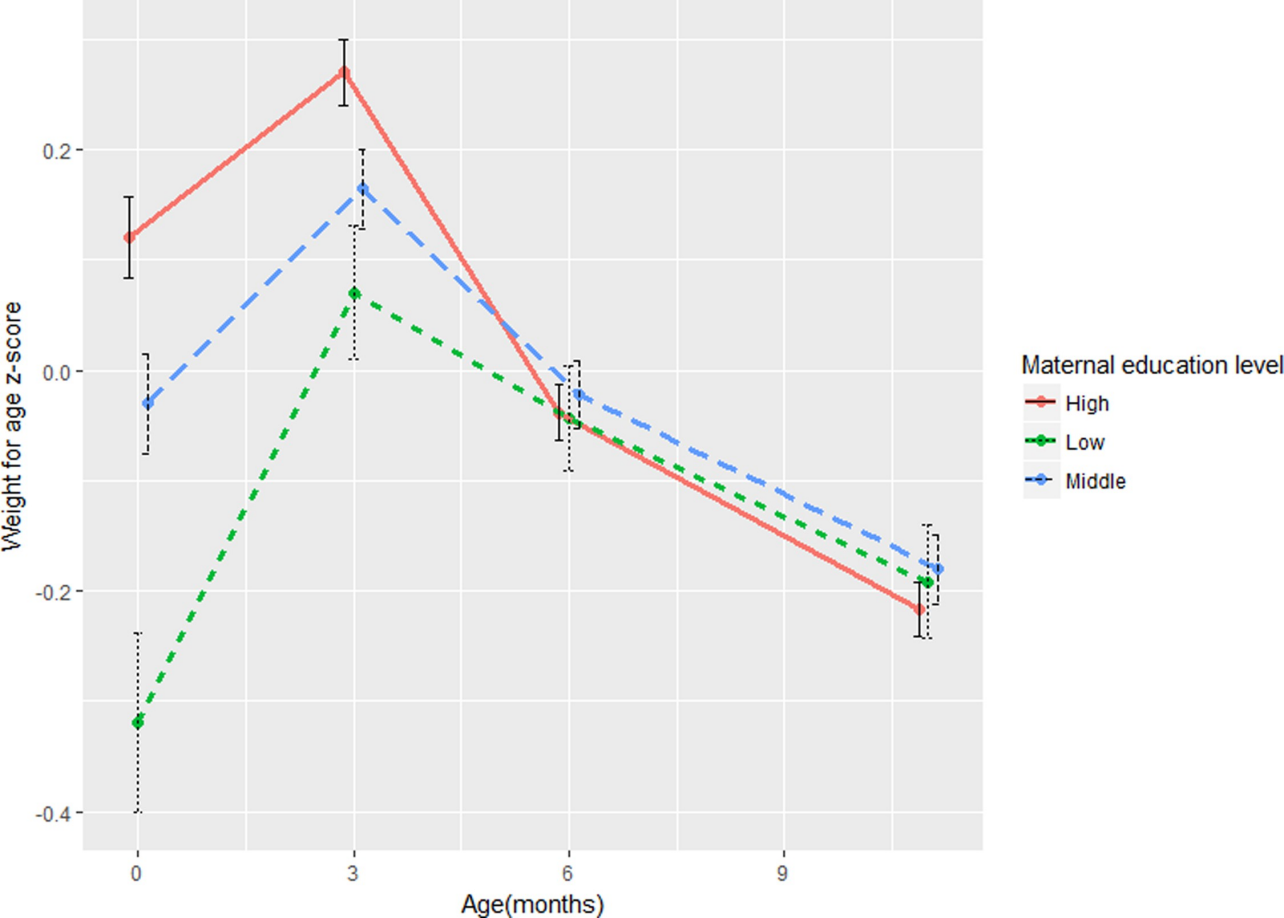
The average weight for age z-score over time from birth to child 12 months according to maternal education level.

Fig 1 shows the patterns of average WAZ over time according to maternal education level. Infants of lower educated mothers had lower weight at birth, and gained weight more rapidly in the first half of infancy. After infant age 6 months, there was no difference in WAZ between maternal education subgroups.

Table 2 shows that infants with low- and middle-educated mothers had greater increase in WAZ in the period of 0–3 months, and 0–6 months compared to infants with high-educated mothers. The differences in WAZ changes between infants of low-educated mothers and those of high-educated mothers in the period of 0–3 months and 0–6 months were 0.24 (95% confidence interval (CI) 0.09–0.38), 0.41 (95% CI 0.27–0.57) respectively. Infants with low-educated mothers also had a higher ratio of rapid weight gain during the period 0–3 months, and 0–6 months. We repeated the analysis by using paternal education level, paternal and maternal employment status as indicators of SES, the results were comparable (S2 Table).

**Table 2:** The association of mother education level with infant weight gain and rapid growth at different time windows.

Age windows	0–3 months	0–6 months	6–12 months
	n = 1661	n = 2002	n = 1848
*Gain in WAZ* ^1^	β (95% CI)	β (95% CI)	β (95% CI)
Mother education level			
Low vs High	0.24(0.09,0.38) ***	0.42(0.27,0.57) ***	0.03(-0.04,0.09)
Middle vs High	0.08(-0.02,0.18)	0.18(0.07,0.28) **	0.03(-0.02,0.07)
*Rapid weight gain (Yes vs No)*	OR (95% CI)	OR (95% CI)	OR (95% CI)
Mother education level			
Low vs High	1.62(1.21,2.16) **	1.87(1.43,2.44) **	1.1(0.64,1.89)
Middle vs High	1.26(1.02,1.55)	1.52(1.25,1.86)	1.14(0.78,1.66)

Note: Models adjusted for exact age at measurements, gender and ethnic background of infants.

*p < 0.017, **p < 0.01, ***p < 0.001.

^1^: Weight for age z-score

### Potential mediators

Table 3 shows the results of multivariate linear regression models for factors associated with weight gain at 0–6 months, factors associated with infant weight gain included infant gender, ethnic background, birth weight, gestational age, maternal gestational weight gain, maternal diabetes, parity, maternal and paternal height, maternal pre-pregnancy BMI, duration of breastfeeding, and age at introduction of complementary foods.

**Table 3:** Factors associated with infant weight gain at different time windows: results from multivariate linear regression models.

Age windows	0–6 months
	β (95%CI)
**Infant characteristics**	
Weight for gestational age z-score	-0.77(-0.81, -0.73) ***
Gestational age at birth (weeks)	-0.37(-0.40, -0.35) ***
**Prenatal factors**	
Maternal age at child birth (years)	-0.01(-0.02, 0.00)
Maternal pre-pregnancy BMI (kg/m2)	0.01(0.00, 0.01) *
Paternal BMI (kg/m2)	0.01(0.00, 0.02)
Maternal height (meters)	0.64(0.12, 1.16) *
Paternal height (meters)	1.16(0.68, 1.64) ***
Gestational weight gain mother (kg)	0.00(0.00, 0.01)
Maternal hypertension	-0.04(-0.15, 0.07)
Maternal diabetes	0.17(-0.09, 0.42)
Parity, primipara	-0.05(-0.13, 0.02)
**Infant feeding practices**	
Breastfeeding duration, (months)	-0.05(-0.06, -0.03) ***
Age at introduction of complementary feeding, (months)	-0.08(-0.12, -0.04) ***

Note: The models included all the potential explanatory variables, and exact age at weight measurement.

*p < 0.05, **p < 0.01, ***p < 0.001

**Fig 2:**
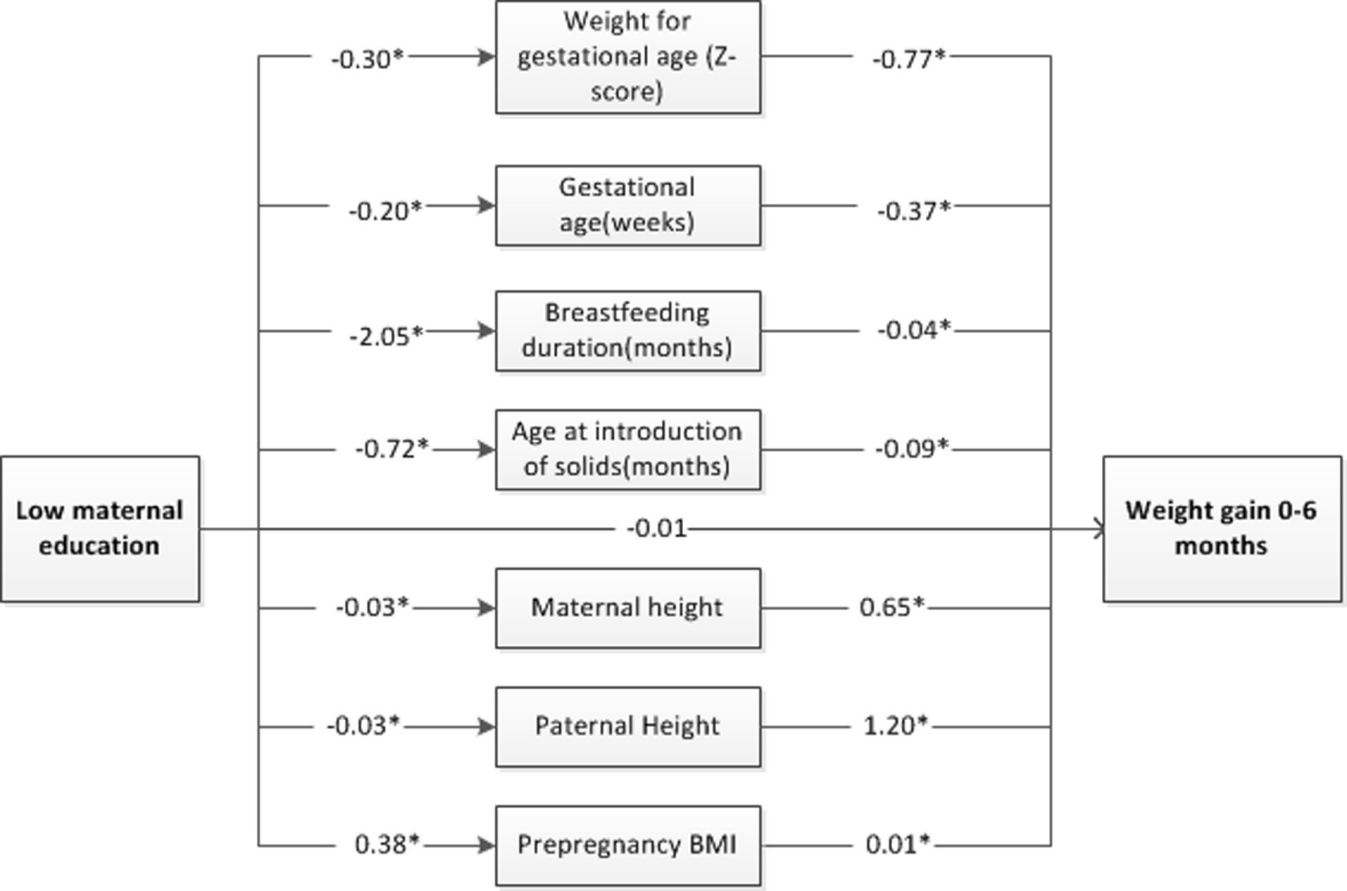
Path analysis model for maternal education and weight gain 0–6 months. Note: *p<0.05.

In the path analysis, we focused on explaining the difference in infant weight gain between low and high maternal education level in the period of 0–6 months. Analyses revealed that a low-maternal education level, compared to a high-maternal education level, was associated with increased weight gain in the first 6 months of infancy indirectly through infant birth weight, infant gestational age, maternal pre-pregnancy BMI, maternal and paternal heights, duration of breastfeeding, and age at introduction of complementary food (Fig 2). Low maternal educational level had no direct effect on infant weight gain in the period of 0–6 months. (Table 4). Infants of mothers with low educational level have increases in WAZ in the period of 0–6 months of 0.23 via weight for gestational age z-score, 0.07 via gestational age, 0.16 via breastfeeding duration and age at introduction of complementary feeding in total. In addition, parental heights have a counter effect (-0.05) on the association between lower maternal educational level and gains in WAZ.

**Table 4:** The pathways linking maternal education level and infant weight gain in the period of 0–6 months.

	Low VS High maternal education level	
	Effects	Proportion mediated
Direct effect	-0.01	-1%
Indirect effect through potential mediators		
Weight for gestational z-score	0.23*	57%
Gestational age	0.07*	18%
Breastfeeding duration	0.09*	22%
Age at introduction of complementary foods	0.07*	17%
Maternal Height	-0.02*	-4%
Paternal Height	-0.03*	-8%
Maternal pre-pregnancy BMI	0.00	1%
Total effect	0.41	

Note: The model adjusted for exact age of the infant, and child ethnic background.

*The effect was statistically significant (*p*<0.05)

## Discussion

Overall, we found clear evidence of a SES gradient in infant weight gain in the period of 0–6 months in this population based sample from the Netherlands. The main mediating factors explaining the association between family SES and infancy weight gain included birth weight, gestational age, and infant feeding practices. After adjusting for all the potential mediators, maternal education level was no longer associated with infant weight gain.

In our study population, infants with lower educated mother had lower birthweight, and gain weight more rapidly in the first 6 months. The associations between low maternal education level and more rapid infant weight gain in the first 6 months could largely be explained by shorter gestational age, and smaller weight for gestational age z-score. In line with previous studies, [36–39] our study found that infants with lower educated mothers had shorter gestational age, and had smaller weight for gestational age *z*-score. Weight gain during the first few months after birth is highly dependent on birth weight, since babies born with a smaller size tend to catch-up, while heavier babies tend to catch-down.[19] Increased weight gain during infancy following low birthweight have been independently related to increased risk of obesity, and cardiovascular risk factors such as hypertension, and diabetes/insulin resistance.[11, 12, 40–43] In addition, studies have also suggested that increased weight gain during infancy is associated with cardiovascular risk factors in later life independent of birthweight.[44]Therefore, the increased weight gain following lower birth weight in the low maternal educational group deserves further attention. However, it should be noted that infant weight in the lower maternal educational group did not exceed that in the higher educational group during infancy. It is possible the increased weight gain in the lower SES group is due to a nature convergence of the infant weight to the average level after birth. Whether the SES divergence in weight gain during infancy can explain the SES inequalities in obesity and cardiovascular risk factors in later life requires further investigation.

In addition to infant birth weight, infant feeding practices including breastfeeding duration and age at introduction of complementary food explained the remaining associations between low maternal education level and increased infant weight gain. The effect explained by infant feeding practices was 40% in total. In previous studies, infant feeding practices explained 62% of the effect in the first 3 months,[15] and 27% of the effect in the first year [16]. Consistent with previous studies, [45–48] we found that lower educated mothers were less likely to initiate breastfeeding, breastfed for a shorter period, and introduced complementary food at an earlier age. Both formula feeding [49, 50] and early introduction of complementary foods [51] have been associated with increased weight gain during infancy. Higher protein and energy content from formula and complementary food [52] may stimulate the secretion of insulin-like growth factor which enhances growth in the first 6 months of infancy.[53] In addition, breastfed infants may learn to self-regulate their intake better than formula fed infants.[54] It should be noted that reverse causalities might exist for the associations of breastfeeding duration and age at introduction of complementary feeding with infant weigh gain. Parents of infants experiencing more rapid growth may stop breastfeeding early and introduce complementary feeding early, because they think their child may need more energy. If a such a reverse causality exists, we may have overestimated the real effect of breastfeeding duration and age at introduction of complementary feeding on infant weight gain. And therefore, the indirect effect of maternal educational level on infant weight gain mediated by breastfeeding duration and age at introduction of complementary feeding may have been overestimated.

Parental heights have counter effects on the association between low maternal education level and weight gain during 0–6 months. On average, parents of higher SES have higher statue. Parental statues can influence growth rate during infancy- infant with taller parents tend to have higher weight and length gain.[20]

Maternal educational level was chosen as the main indicator of family SES in the present study, as healthy infant weight development is mainly related to maternal related factors, such as child birthweight, maternal BMI, and infant feeding. In addition, educational level is a stable variable accomplished in early adult life, and is relevant to people regardless of age and working circumstances. We repeated our analysis using other family SES indicators, including paternal educational level, maternal employment status and paternal employment status. In addition, we combined maternal and paternal educational level as parental educational level, which was defined as the highest attained educational level of both parents. As we expected, paternal educational level had similar (yet weaker) graded association with infant weight gain than low maternal educational level (S3 Table). The association of parental educational level with infant weight gain was similar to maternal educational level and tend to be stronger. This might indicate that the more unfavorable socioeconomic conditions a family have, the stronger the effect on infant weight gain. The association between maternal unemployment and infant weight gain could be partly explained by maternal educational level. Also, unemployed mothers may have more time to feed their children and therefore contribute to higher BMI of children. The absence of association between paternal employment status and infant weight gain might be due to the low percentage of paternal unemployment (3%), and the instability of employment status

In addition of infant weight for age z-scores, weight-for-length z-scores and BMI for age z scores have also been relevant to the development of body composition. We tested the association between family SES and the development of weight-for-length z-scores and BMI z-scores of the children in their first year of life. Maternal educational level was associated with higher increases in BMI z-scores between age 0 to 6 months, while was not significantly associated with changes in weight for length z-scores. This might be because weight for length z-scores are not age adjusted, and may be not sensitive to changes in infant weight gain in a short period. Previous studies indicated that weight for length z-scores may lack reliability in younger children.

The present study has a number of strengths. Firstly, the repeated measurements of infant weight allowed us to track the trajectory of infant weight gain. Secondly, we used a literature and hypothesis driven approach to select relevant variables. All mediating variables were selected based on a priori association with infant weight changes. The association between SES and increased infant weight gain was fully explained by the selected potential confounding factors. Thirdly, by using path analysis, correlations between mediators were taken into account, and we were able to calculate the independent effect mediated by multiple mediators’.[33] Some limitations have to be mentioned as well. Firstly, selective participation and loss to follow-up is possible, especially among the lower-educated groups. However, this may not bias our finding, as the association between exposure and the outcome will only be biased when participation is associated with both exposure and outcome. Secondly, we were not able to assess the effect of maternal smoking on infant weight gain which may have explained the association between maternal education and infant weight gain.[16] Thirdly, we used data from a cluster randomized controlled trial for childhood overweight prevention. It is possible that interventions are more effective when maternal educational levels are higher, as high educated mothers have higher receptiveness to healthy education messages. Therefore, intervention might strengthen the maternal educational gradients in infant weight gain. However, our interaction analyses suggested that the SES difference does not differ by the intervention groups. Also, sensitivity analysis using data from children who did not received any interventions suggested that the association between maternal educational level and infant weight gain was comparable to the results using all the available subjects. [55]

In conclusion, our findings indicate that infants from lower SES families have more rapid weight gain during the first half of infancy than those from higher SES families. The association between lower SES and more rapid weight gain can be explained by lower birthweight, shorter gestational age, shorter duration of breastfeeding, and earlier introduction of complementary foods. Promotion of a healthy pregnancy, optimizing duration of breastfeeding and timing of complementary feeding, in particular among low educated women, may contribute to normalizing infant growth and reduce adverse consequences of increased infant weight gain.

## Supporting information

S1 Table The measurement and recoding method for breastfeeding duration and age at introduction of complementary feeding. (DOCX)

S2 Table The distribution of the potential mediators in the observed dataset and in one of the 20 imputed datasets. (DOCX)

S3 Table The association of different indicators of socioeconomic status with infant weight gain at different time windows. (DOCX)

S4 Table The association of maternal educational level with gains in weight for height z-score (WHZ) and gains in BMI for age z-score (BMIZ) at different time windows. (DOCX)

S5 Table The association of maternal educational level with infant weight gain at different time windows in children received no interventions. (DOCX)

S6 Table Factors associated with infant weight gain in the period of 0–6 months: results from multivariate linear regression models- complete case analysis. (DOCX)

S7 Table The pathways linking maternal education level and infant weight gain in the period of 0–6 months- complete case analysis. (DOCX)
