# Supplementary material for: Relationship between socioeconomic status and weight gain during infancy: The BeeBOFT study
Source: PLoS One. 2018 Nov 2;13(11):e0205734. doi: 10.1371/journal.pone.0205734 (PMC6214496; doi:10.1371/journal.pone.0205734)
Supplement: S1 Table — (DOCX) [file pone.0205734.s001.docx]

Table S1. The measurement and recoding method for breastfeeding duration and age at introduction of complementary feeding

| Variables | Questions | Answering categories and Recoding |
| --- | --- | --- |
| Breastfeeding duration (months) |  |  |
|  | Did you started breastfeeding after child birth | No🡺 0  Yes🡺 recoding according to the following questions |
|  | How old was your child when you stopped breastfeeding totally? | 1 I am still giving breastfeeding 🡺6.5  2 within 1 week 🡺 0.2  3 In week 2🡺0.5  4 In week 3🡺0.8  5 In week 4🡺 1  6 1~2 months🡺 1.5  7 2~3 months🡺 2.5  9 3~4 months🡺 3.5  10 4~5 months🡺 4.5  11 >5 months 🡺 5.5 |
| Age at introduction of complementary feeding (months) |  |  |
|  | Please indicate how old was your child when you gave each of the following product for the first time respectively?  A list of food were provided, including (syrup or instant lemonade, diet soda, sweet dairy drinks, milk/buttermilk, custard/yogurt/curd, pap in a bottle, pap from a plate, bread without toppings, bread with toppings, baby biscuits, chocolate or candy, crackers or grissini, fruit from jars, fresh fruit, vegetable jars without fish or meat, vegetables jars with fish or meat, pasta/rice/potato, fresh vegetable, fish/meat/meat substitutes.) | 1~2 months🡺 1.5  2~3 months🡺 2.5  3~4 months🡺 3.5  4~5 months🡺 4.5  >5 months 🡺 5.5  Never🡺 6.5  The timing of introduction of complementary feeding was defined as the youngest age of the infant when any of the above mentioned complementary food were introduced to the infant. |
