# Supplementary material for: Relationship between socioeconomic status and weight gain during infancy: The BeeBOFT study
Source: PLoS One. 2018 Nov 2;13(11):e0205734. doi: 10.1371/journal.pone.0205734 (PMC6214496; doi:10.1371/journal.pone.0205734)
Supplement: S2 Table — (DOCX) [file pone.0205734.s002.docx]

Table S2 The distribution of the potential mediators in the observed dataset and in one of the 20 imputed datasets.

|  | Observed data | | Imputed data | |
| --- | --- | --- | --- | --- |
|  | Mean | SD | Mean | SD |
| Gestational age at birth (weeks) | 39.66 | 1.30 | 39.66 | 1.29 |
| Weight for gestational age z-score | 0.05 | 1.01 | 0.05 | 1.01 |
| Maternal age at child birth (years) | 30.90 | 4.29 | 30.89 | 4.29 |
| Maternal height (meters) | 1.70 | 0.07 | 1.70 | 0.07 |
| Paternal height (meters) | 1.83 | 0.07 | 1.83 | 0.07 |
| Maternal pre-pregnancy BMI (kg/m2) | 24.27 | 4.53 | 24.28 | 4.53 |
| Paternal BMI (kg/m2) | 25.24 | 3.31 | 25.23 | 3.31 |
| Breastfeeding duration, (months) | 2.82 | 2.78 | 2.85 | 2.77 |
| Age at introduction of complementary feeding, (months) | 4.61 | 0.95 | 4.58 | 0.94 |
| Gestational weight gain mother (kg) | 14.14 | 5.29 | 14.09 | 5.28 |

Note: we imputed factors associated with infant weight gain in the period of 0-6 months.
