# Supplementary material for: Relationship between socioeconomic status and weight gain during infancy: The BeeBOFT study
Source: PLoS One. 2018 Nov 2;13(11):e0205734. doi: 10.1371/journal.pone.0205734 (PMC6214496; doi:10.1371/journal.pone.0205734)
Supplement: S3 Table — (DOCX) [file pone.0205734.s003.docx]

Table S3. The association of different indicators of socioeconomic status with infant weight gain at different time windows.

|  | 0-3 months | 0-6 months | 6-12 months |
| --- | --- | --- | --- |
|  | n=1661 | n=2002 | n=1848 |
| *Gain in weight for age SDS* | *β* (95% CI) | *β* (95% CI) | *β* (95% CI) |
| Paternal education level |  |  |  |
| Low vs High | 0.20(0.07,0.33) ^**^ | 0.34(0.20,0.48)^***^ | 0.05(-0.01,0.11) |
| Middle vs High | 0.12(0.01,0.22) ^*^ | 0.18(0.07,0.29) ^**^ | 0.02(-0.03,0.07) |
| Maternal employment status |  |  |  |
| Without paid job vs with paid job | 0.20(0.07,0.33) ^**^ | 0.27(0.13,0.40) ^***^ | -0.02(-0.08,0.04) |
| Paternal employment status |  |  |  |
| Without paid job vs with paid job | 0.18(-0.07,0.44) | 0.11(-0.18,0.40) | 0.00(-0.12,0.13) |
| Parental educational level |  |  |  |
| Low vs High | 0.23(0.06,0.39) | 0.45(0.27,0.63) ^***^ | 0.08(0.01,0.16) |
| Middle vs High | 0.12(0.02,0.22) | 0.25(0.14,0.36) ^**^ | 0.03(-0.02,0.08) |

Note: The models were adjusted for child gender, ethnic background, age at weight measurement, and intervention group.

^*^p < 0.017, ^**^p < 0.01, ^***^p < 0.001.
