# Supplementary material for: Relationship between socioeconomic status and weight gain during infancy: The BeeBOFT study
Source: PLoS One. 2018 Nov 2;13(11):e0205734. doi: 10.1371/journal.pone.0205734 (PMC6214496; doi:10.1371/journal.pone.0205734)
Supplement: S4 Table — (DOCX) [file pone.0205734.s004.docx]

Table S4. The association of maternal educational level with gains in weight for height z-score (WHZ) and gains in BMI for age z-score (BMIZ) at different time windows

| Age windows | 0-3 months | 0-6 months | 6-12 months |
| --- | --- | --- | --- |
| *Outcome: Gain in WHZ* | β (95% CI) | β (95% CI) | β (95% CI) |
| Mother education level |  |  |  |
| Low vs High | -0.01(-0.19,0.18) | 0.02(-0.15,0.19) | -0.05(-0.13,0.03) |
| Middle vs High | -0.06(-0.19,0.07) | -0.01(-0.13,0.11) | -0.03(-0.09,0.02) |
| *Outcome: Gain in BMIZ* |  |  |  |
| Mother education level |  |  |  |
| Low vs High | 0.17(-0.06,0.40) | 0.29(0.07,0.50) ^*^ | -0.06(-0.14,0.03) |
| Middle vs High | 0.00(-0.16,0.16) | 0.11(-0.04,0.26) | -0.04(-0.10,0.02) |

Note: The models were adjusted for child gender, ethnic background, age at weight measurement, and intervention group.

^*^p < 0.017
