# Supplementary material for: Relationship between socioeconomic status and weight gain during infancy: The BeeBOFT study
Source: PLoS One. 2018 Nov 2;13(11):e0205734. doi: 10.1371/journal.pone.0205734 (PMC6214496; doi:10.1371/journal.pone.0205734)
Supplement: S5 Table — (DOCX) [file pone.0205734.s005.docx]

Table S5. The association of maternal educational level with infant weight gain at different time windows in children received no interventions

| Age windows | 0-3 months |  | 0-6 months |  | 6-12 months |  |
| --- | --- | --- | --- | --- | --- | --- |
| *Gain in WAZ ^1^* | *β* (95% CI)^1^ | p-value^2^ | *β* (95% CI) ^1^ | p-value^2^ | *β* (95% CI) ^1^ | p-value^2^ |
| Mother education level |  |  |  |  |  |  |
| Low vs High | 0.21(0.03,0.39) | 0.57 | 0.42(0.23,0.60) | 0.71 | 0.02(-0.06,0.10) | 0.66 |
| Middle vs High | 0.11(-0.01,0.23) | 0.54 | 0.23(0.10,0.36) | 0.26 | 0.01(-0.05,0.07) | 0.31 |

^1^ :The models were adjusted for child gender, ethnic background, and age at weight measurement.

^2^ : The p-values for the interaction terms of SES indicators and intervention group in the models predicting infant weight gain at different time windows
