# Supplementary material for: Relationship between socioeconomic status and weight gain during infancy: The BeeBOFT study
Source: PLoS One. 2018 Nov 2;13(11):e0205734. doi: 10.1371/journal.pone.0205734 (PMC6214496; doi:10.1371/journal.pone.0205734)
Supplement: S6 Table — (DOCX) [file pone.0205734.s006.docx]

Table S6. Factors associated with infant weight gain in the period of 0-6 months: results from multivariate linear regression models- complete case analysis.

| Age windows | 0-6 months |
| --- | --- |
|  | β (95%CI) |
| **Infant characteristics** |  |
| Weight for gestational age z-score | -0.76(-0.80,-0.72) ^***^ |
| Gestational age at birth (weeks) | -0.37(-0.40,-0.34) ^***^ |
| **Prenatal factors** |  |
| Maternal age at child birth (years) | 0.00(-0.01,0.01) |
| Maternal pre-pregnancy BMI (kg/m2) | 0.01(0.00,0.01) ^*^ |
| Paternal BMI (kg/m2) | 0.01(0.00,0.02) |
| Maternal height (meters) | 0.74(0.13,1.36) ^*^ |
| Paternal height (meters) | 1.26(0.70,1.82) ^***^ |
| Gestational weight gain mother (kg) | 0.01(0.00,0.02) |
| Maternal hypertension | -0.03(-0.16,0.10) |
| Maternal diabetes | 0.17(-0.19,0.52) |
| Parity, primipara | -0.07(-0.15,0.01) |
| **Infant feeding practices** |  |
| Breastfeeding duration, (months) | -0.05(-0.07,-0.04) ^***^ |
| Age at introduction of complementary feeding, (months) | -0.07(-0.11,-0.02) ^**^ |

Note: The models were adjusted for maternal educational level, child gender, ethnic background, age at weight measurement, and intervention group.^*^p < 0.05, ^**^p < 0.01, ^***^p < 0.001
