# Supplementary material for: Relationship between socioeconomic status and weight gain during infancy: The BeeBOFT study
Source: PLoS One. 2018 Nov 2;13(11):e0205734. doi: 10.1371/journal.pone.0205734 (PMC6214496; doi:10.1371/journal.pone.0205734)
Supplement: S7 Table — (DOCX) [file pone.0205734.s007.docx]

Table S7 The pathways linking maternal education level and infant weight gain in the period of 0-6 months- complete case analysis.

|  | Low VS High maternal education level | |
| --- | --- | --- |
|  | effect | Proportion mediated |
| Direct effect | 0.01 | 2% |
| Indirect effect through potential mediators |  |  |
| Weight for gestational Z-score | 0.22^*^ | 57% |
| Gestational age | 0.05^*^ | 14% |
| Breastfeeding duration | 0.08^*^ | 20% |
| Age at introduction of complementary foods | 0.07^*^ | 17% |
| Maternal Height | -0.02^*^ | -5% |
| Paternal Height | -0.03^*^ | -9% |
| Maternal pre-pregnancy BMI | 0.01 | 4% |
| Total effect | 0.39 |  |

Note : The model adjusted for exact age of the infant, and child ethnic background.

^*^The effect was statistically significant (*p*<0.05)
